# Neural Oscillations in the Somatosensory and Motor Cortex Distinguish Dexmedetomidine‐Induced Anesthesia and Sleep in Rats

**DOI:** 10.1111/cns.70262

**Published:** 2025-02-18

**Authors:** Dengyun Ge, Chuanliang Han, Chang Liu, Zhiqiang Meng

**Affiliations:** ^1^ Shenzhen Key Laboratory of Drug Addiction, Shenzhen–Hong Kong Institute of Brain Science, Shenzhen Institute of Advanced Technology Chinese Academy of Sciences Shenzhen China; ^2^ School of Biomedical Sciences and Gerald Choa Neuroscience Institute The Chinese University of Hong Kong Hong Kong SAR China; ^3^ CAS Key Laboratory of Brain Connectome and Manipulation Chinese Academy of Sciences Shenzhen China

**Keywords:** anesthesia, coherence, dexmedetomidine, motor cortex, sleep, somatosensory cortex

## Abstract

**Background:**

Anesthesia is featured by behavioral and physiological characteristics such as decreased sensory and motor function, loss of consciousness, etc. Some anesthetics such as dexmedetomidine (DEX), induce electroencephalogram signatures close to non‐rapid eye movement sleep. Studies have shown that sleep is primarily driven by the activation of subcortical sleep‐promoting neural pathways.

**Aims:**

However, the neuronal level electrophysiology features of anesthesia and how they differ from sleep is still not fully understood.

**Materials and Methods:**

In the present study, we recorded neuronal activity simultaneously from somatosensory cortex (S1) and motor cortex (M1) during awake, sleep, and DEX‐induced anesthesia in rats.

**Results:**

The results show that DEX increased local field potential (LFP) power across a relatively wide band (1–25 Hz) in both S1 and M1. The coherence between S1 LFP and M1 LFP increased significantly in the delta and alpha bands. Power spectrum analysis during DEX‐induced anesthesia revealed relatively high power in the delta and alpha bands, but low power in the theta and beta bands. Overall, the firing rate of individual neurons decreased after DEX. Correlation analysis of firing rate and LFP power indicate that more neurons were correlated, either positively or negatively, with LFPs during DEX‐induced anesthesia compared to sleep.

**Discussion:**

Although these results showed enhancement of cortical LFP power in both DEX‐induced anesthesia and sleep, different patterns of spike‐field correlation suggest that the two states may be regulated by different cortical mechanisms.

**Conclusion:**

Distinguishing anesthesia from sleep with neural oscillations could lead to more personalized, safer, and more effective approaches to managing consciousness in medical settings, with the potential for broad applications in neuroscience and clinical practice.

## Graphical abstract

By recording neuronal activity from the primary somatosensory cortex and motor cortex simultaneously, during dexmedetomidine (DEX)‐induced anesthesia or sleep, we investigated the cortical response and oscillation properties across different brain areas. Correlation analysis between single neuron activity and LFP power indicates that more neurons were correlated, either positively or negatively, with LFPs during DEX‐induced anesthesia compared to sleep.

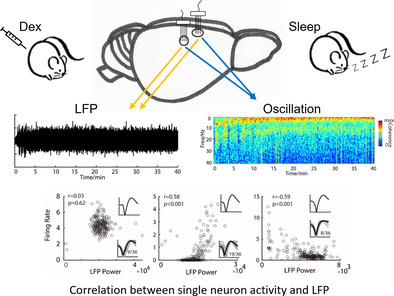

## 1 Introduction

Since the introduction of modern anesthesia, research has been initiated to investigate the underlying mechanisms. It is believed that anesthetics produce a widespread neural inhibition in the central nervous system some of them also activate non‐rapid eye movement (NREM) sleep promoting neuronal populations [1, 2, 3, 4]. Several molecular targets of general anesthetics have been identified over the past decades, but the neural circuits underlying the discrete end points of anesthesia remain incompletely understood [5, 6]. Given the common behavioral features of sleep and anesthesia such as immobility and unconsciousness, there has been growing interest in studying how anesthetics converge onto subcortical sleep‐promoting neural pathways to produce anesthesia in recent years [7, 8, 9, 10]. However, the neural response in the cortex during natural sleep and anesthesia remains to be elucidated.

Previous clinical studies mainly utilized electroencephalogram (EEG) to monitor the dynamics of cortical activity during anesthesia and sleep [11, 12]. During sleep, the EEG signals show specific patterns in different stages. Start at the beginning, high‐frequency (beta, gamma, and alpha), low‐voltage waves will gradually switch to higher voltage, slower waves (theta and delta), which are the feature patterns of NREM sleep [13, 14, 15]. During NREM sleep, stages 1, 2, and 3 are classified depending on the EEG frequency and specific pattern. Spindles or bursting firing of neurons were found shortly after falling asleep. During rapid eye movement (REM) sleep, the EEG patterns were similar to those during awake [13]. In rodent studies, compared to humans, rats exhibit a more fragmented sleep pattern, with most research primarily focusing on rapid eye movement (REM) and non‐rapid eye movement (NREM) sleep stages [16, 17].

Generally, different anesthetics induce unique EEG patterns depending on their molecular targets and pharmacokinetic properties [18]. Some EEG features were found under DEX anesthesia such as increased slow‐delta oscillations across the scalp and increased frontal spindle oscillations which closely approximate the dynamics during NREM sleep [9, 19]. Dexmedetomidine (DEX) is one of the few anesthetics which induce EEG patterns closely approximate NREM sleep in both human and animals [9, 20, 21, 22, 23, 24]. However, EEG signal represents the sum of activities from a large number of neurons [25, 26, 27, 28]. How DEX acts on single neurons to lead to these EEG features remains incompletely understood. Recently, spike‐field coherence is increasingly studied to understand neural coordination, especially in linking local spiking activity with broader local field potentials (LFPs), which aids in analyzing brain communication and cognitive functions [29, 30, 31]. This relationship can reveal functional insights, but also not fully understood in comparing different states.

In the present study, we recorded single neuron activity and LFP simultaneously to study the coherence between these two types of neuroelectrophysiology signals during DEX‐induced anesthesia. In addition, how these signals differ from sleep was compared. The recordings were from primary motor cortex (M1) and primary somatosensory cortex (S1). The S1 and M1 were specifically chosen for the study because these regions have well‐documented roles in sensory processing, movement, and cortical arousal, which are closely linked to states of consciousness. These two brain areas which sends motion signals and receive sensory inputs, respectively, were probably affected the most during anesthesia [32, 33].

The results show that, DEX‐induced anesthesia was accompanied by a decrease in single neuronal activity and an increase in LFP power spectra. Although these were similar to what was seen during NREM sleep, more neurons were found to be correlated, either positively or negatively, to the corresponding LFPs during DEX‐induced anesthesia compared to sleep. In addition, significant increase in LFP coherence of the delta and low alpha band between S1 and M1 was found during DEX anesthesia but not NREM sleep. These results may contribute to further understanding on the cortical mechanism of DEX anesthesia.

## 2 Materials and Methods

### 2.1 Animal Preparation

Adult male Sprague–Dawley rats (Charles River Laboratories, China) were fed ad libitum and housed in rooms with a 12‐h light–dark cycle, with lights on at 8 am and off at 8 pm. All cage components, including bedding, cage furnishings, and enrichment were autoclaved before use. The room environment was maintained at 68°F to 72°F (20°C–24°C), 40% to 70% humidity. All surgical and experimental protocols were approved by the Institutional Animal Care and Use Committees at the Shenzhen Institute of Advanced Technology, Chinese Academy of Sciences.

### 2.2 Surgery and Electrodes Implantation

**FIGURE 1:**
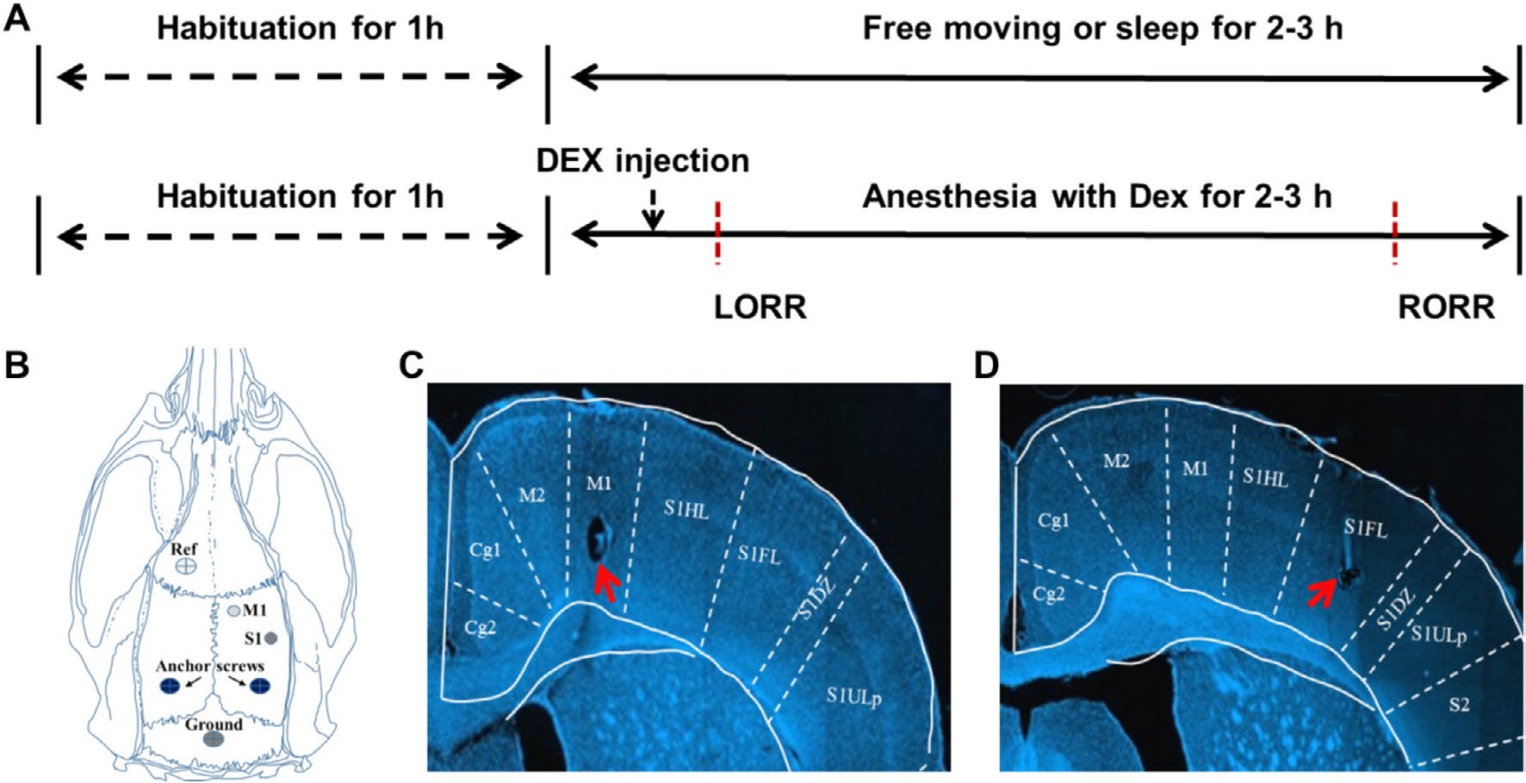
Experiment design and electrophysiology recording sites. Experiment protocol (A). Schematic of the M1 and S1 microelectrodes implantation, reference and ground electrodes, and bone screw sites on an overhead view of the rat skull (B). Lesion site (red arrows) to confirm the electrophysiological recording area in M1 (C) and S1(D). LORR, Loss of righting reflex; RORR, Return of righting reflex.

All procedures were carried out in a sterile laboratory. Rats (9 weeks old at the time of surgery) were anesthetized using 3% isoflurane followed by maintenance at 2% isoflurane (Cat#: R510‐22‐10, RWD Life Science Co., China). Then, they were placed in a stereotaxic frame (Model H1655101, RWD Life Science, China). The animal's body temperature was maintained at 37°C using a heating pad (50‐7220F, Harvard Apparatus, USA). After opening the skin and clearing connective tissue from the top of the skull, craniotomies were made unilaterally over the primary motor and primary somatosensory cortex with a microdrill (Cat#: 78001, RWD Life Science, China; see Figure 1B for a schematic of craniotomies). Bregma was used as the reference for electrodes coordinates. The coordinates for somatosensory cortex and motor cortex were AP: −2.16 mm; ML: +4 mm; DV: −1.6 mm and AP: +0.96 mm; ML: +2.0 mm; DV: −1.6 mm, respectively. Four tetrodes were implanted into each recording site. The tetrodes were made with a wire twister in the laboratory. Each tetrode consisted of four wires twisted together. The wires were purchased from the California Fine Wire Company (Platinum 20% iridium, 0.0007 in. diameter, Cat#: CFW2025853, California Fine Wire). Two anchor screws were used to hold the dental cement and electrodes on the skull. Two other stainless screws at the posterior and anterior region of the skull served as the ground and reference, respectively. After surgery, rats were given antibiotic (Gentamicin, 50 mg/kg; QUANYU, Shanghai, China) and singly housed in home cages. The cup‐handling procedure was performed twice a day (5 min each session) at least 3 days before experiments to minimize potential stress [34]. Rats were transferred to the testing room using their home cages at least 1 h before experiments.

### 2.3 Experiment Design

Rats with pre‐implanted electrodes were transferred to the experimental room using their individual home cage. The habituation time before each recording was at least 1 h. White noise (50 dB SPL) was applied throughout the recording session. Before the experiment, a digital headstage was plugged into the connector of the electrodes for electrophysiological recording. The behaviors were recorded by an overhead camera. The experiment protocol for recordings during DEX‐induced anesthesia and sleep status is shown in Figure 1A. Briefly, the recordings were carried out on two consecutive days. On day 1, the rats were left freely moving within their individual cages during simultaneous electrophysiology and behavior recordings. The wake and sleep status were classified by the behavior and the corresponding local filed potential signature (for details please see the Classification of sleep stages section). On day 2, after 30 min of baseline recording, anesthesia was induced by DEX (0.05 mg/kg, 0.2 mg/kg, i.p. Cat#: 138445‐3, Orion Pharma, Finland) [35, 36, 37]. The recording continued until the regain of righting reflex. The behavior of the animals was recorded and tracked using EthoVision XT software (Noldus, Netherland). The moving speed of the animal was calculated from the tracking files and smoothed using a LOWESS method [38].

### 2.4 Electrophysiological Recordings

Neural activity from S1 and M1 was recorded simultaneously throughout awake, sleep, and DEX‐induced anesthesia. During recording, raw signals were sampled at 40 kHz (OmniPlex, Plexon). Single neuron spikes and LFPs are sampled and filtered from raw signals offline later on. Spikes were sampled at 40 kHz with a bandpass filter from 400 Hz to 7 kHz. LFPs were sampled at 1 kHz with a bandpass filter from 0.7 Hz to 300 Hz. At the end of the experiments, electrolytic lesions were made around the electrode tip to verify the recording sites (Figure 1C,D). All recordings were performed during the lights‐on hours. A total of 10 male rats received tetrode implantation for in vivo recording. A total of 50 min's data (10 min wakefulness, 30 min NREM sleep, and 10 min REM sleep) was included for the data analysis.

### 2.5 Spike Sorting

Spikes were sorted using waveform principal component analysis (Offline Sorter; Plexon). The spiking activity was obtained by applying a minimum threshold of 5 standard deviations (SDs) to exclude background noise from the raw voltage tracings on each channel. Only spikes that were stable through the wake period and showed recovery when the rats regain righting reflex were included in the analysis.

### 2.6 Classification of Sleep Stages

The rats were first classified into active and inactive states depending on their moving speed in 10s epochs (active state: moving speed > 0.5 cm/s, inactive state: moving speed < 0.5 cm/s). Sleep stages were further determined only within the inactive states. More specifically, different stages of sleep were determined based on the power ratio of theta band (4–7 Hz) and delta band (1–4 Hz) from the local filed potential activity during the inactive period. As was used in previous studies [39, 40], NREM sleep was defined as continuous high amplitude of delta‐band activity (1–4 Hz), whereas REM sleep was defined as high amplitude of theta‐band activity (4–7 Hz). During natural sleep state, rats displayed behavioral phenotypes of curled body posture and eyelid closure that is typical of previously reported normal sleep [41].

### 2.7 Power Spectrum Analysis

The LFP data during awake, sleep, and DEX‐induced anesthesia was determined in 10s epochs. Each epoch was conducted with power spectrum analysis, which is a technique for decomposing complex signals into simpler signals based on the Fourier transform (Equations 1 and 2). The LFP power spectra was normalized to the baseline before averaging data from all rats. The power spectral density (PSD) for LFP data was computed using the multi‐taper method (TW = 3, *K* = 5 tapers, Chronux toolbox (http://chronux.org/)). Analysis was conducted using custom‐written and existing functions in MATLAB (The Math Works). Similar methods have been used in previous biological research [42, 43, 44, 45, 46]. Five frequency bands were used in this study, delta, 1–4 Hz, theta, 4–7 Hz, alpha, 8–13 Hz, low beta, 15–20 Hz, high beta, 20–25 Hz.

(1) $$X\left(\omega \right)=\int_{-\infty }^{\infty }x\left(t\right)e^{-i\mathrm{\omega t}}\mathrm{dt}$$

(2) $$\mathrm{PSD}\left(f\right)=\lim_{T\to \infty }\frac{1}{T}{\left|\int_{-\infty }^{\infty }x\left(t\right)W_{T}\left(t\right)e^{-i\mathrm{\omega t}}\mathrm{dt}\right|}^{2}$$

where $W_{T}\left(t\right)$ is unity within the arbitrary period and zero elsewhere, *T* is centered about some arbitrary time *t* = *t*_0_.

### 2.8 Coherence Analysis

We evaluated field‐field coherence Cxy by calculating the coherency between different recording sites (e.g., *x* in M1 and *y* in S1) as the cross‐spectra Sxy between signals in *x* and *y*, normalized by the geometric mean of their autospectra (Sxx and Syy) (Equation 3). This was estimated using the multi‐taper method (time‐bandwidth product, 3; tapers, 5; Chronux toolbox (http://chronux.org)) and implemented using custom software written in MATLAB. Similar methods have been used in previous biological research [29].

(3) $$\mathrm{Cxy}\left(f\right)=\frac{\mathrm{Sxy}\left(f\right)}{\sqrt{\mathrm{Sxx}\left(f\right)\mathrm{Syy}\left(f\right)}}$$

### 2.9 Statistical Analysis

Statistical analysis was performed using MATLAB. A normality test was performed on each dataset using the Shapiro–Wilk test or D′ Agostino and Pearson test. For comparison of two group means, parametric tests (paired *t*‐test or unpaired *t*‐test) were used if the dataset was normally distributed; otherwise, nonparametric tests (Wilcoxon signed rank test or Mann–Whitney U test) were used. We conducted an independent *t*‐test comparing the difference of LFP power in M1 and S1 in different frequency bands. Pairwise *t*‐test was conducted comparing the difference of LFP power before and after the injection of DEX, and the difference of coherence between M1 and S1 before and after the injection of DEX. One‐way ANOVA was used to compare the difference among the LFP power in state of DEX‐induced anesthesia, REM sleep, and NREM sleep.

## 3 Results

### 3.1 DEX Increased in LFP Power Lies Across Multiple Frequency Bands in Both S1 and M1

**FIGURE 2:**
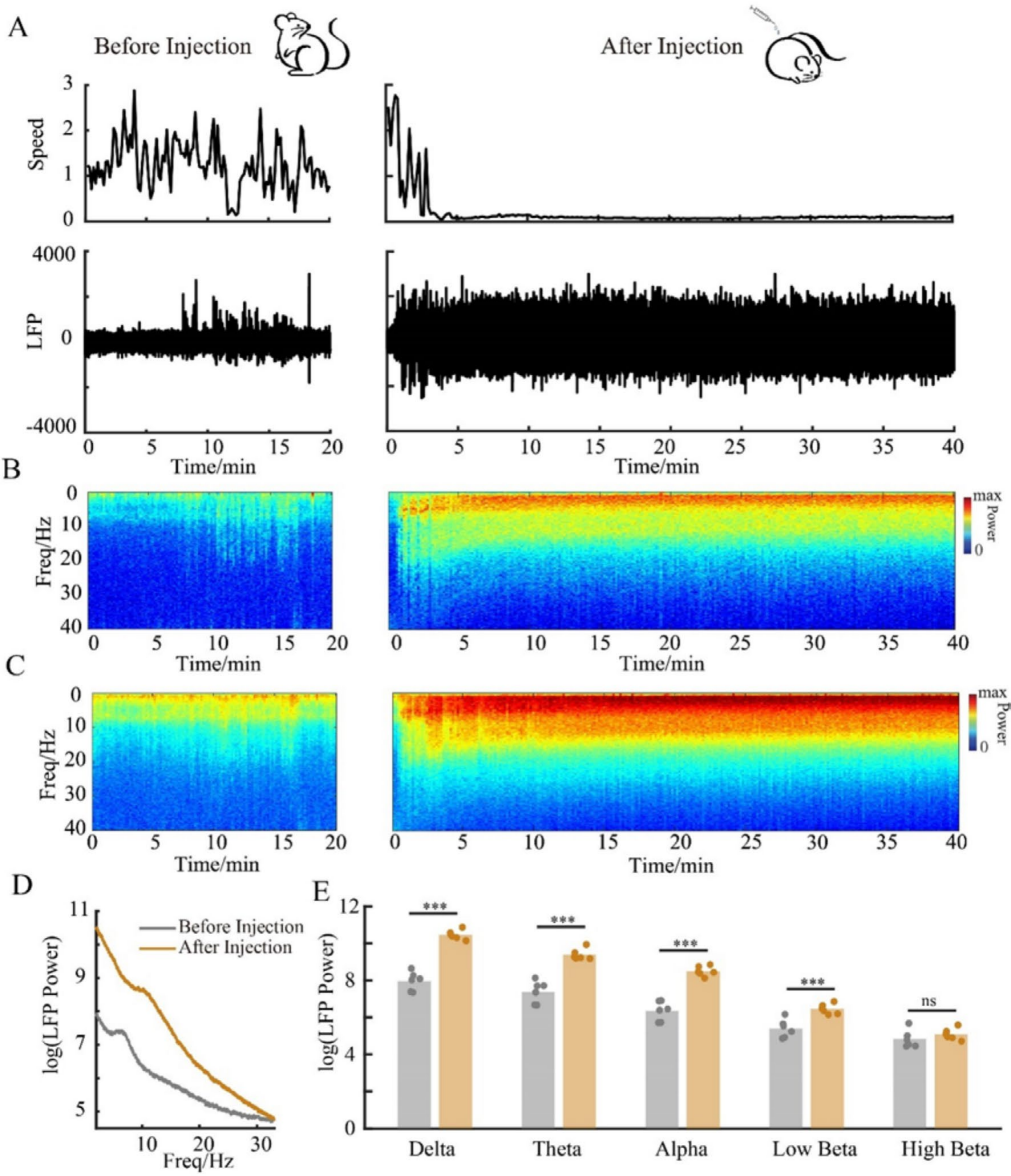
Spectrograms of S1 and M1, and spectral analysis comparing free moving (awake) to DEX‐induced anesthesia. (A) A representative of rat moving speed (top) and LFP (bottom, data is from M1) before and after DEX injection. (B) Spectrogram of one rat before and after DEX injection. (C) Group spectrograms of six rats before and after DEX injection. (D and E) Comparison of LFP power by different frequency bands before and after DEX injection. ***p < 0.001. Five frequency bands were used in this study, delta, 1–4 Hz, theta, 4–7 Hz, alpha,8–13 Hz, low beta, 15–20 Hz, high beta, 20–25 Hz.

Figure 2A shows the moving speed of the rats before and after DEX injection (0.2 mg/kg). DEX induced lying motionless in rats accompanied with loss of righting reflex. The pattern of cortical activity under DEX‐induced anesthesia was primarily featured by increased power in low frequency band (Figure 2B–E). Similar to 0.2 mg/kg DEX, 0.05 mg/kg DEX increased LFP power in M1 at the theta, alpha, and low beta bands. The only difference is lower dose DEX did not affect the delta band significantly (data not shown). Data was from both S1 and M1 because no significant difference in terms of the power spectrum was found between S1 and M1 (Figure S1A,B). LFP examples of wakefulness, NREM sleep, REM sleep, and DEX‐induced anesthesia are shown in Figure S2.

### 3.2 DEX‐Induced Anesthesia Was Characterized by Significantly Higher Oscillatory Coherence in Delta and Alpha Band Between S1 and M1

**FIGURE 3:**
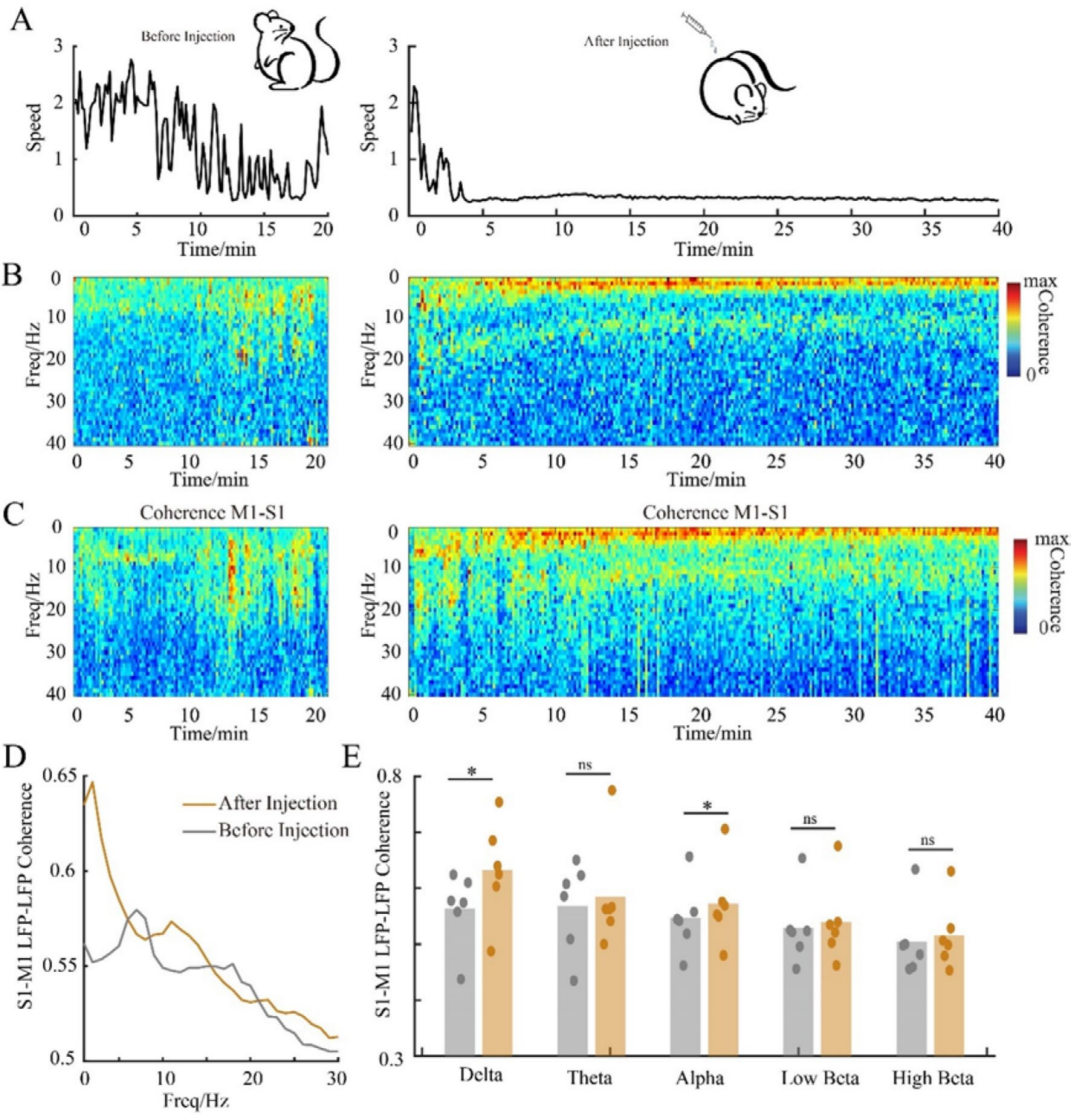
Coherograms between S1 and M1 during DEX‐induced anesthesia. (A) The locomotor activity of the rats. (B) DEX‐induced coherograms change between S1 and M1 from one rat. (C) DEX‐induced coherograms change between S1 and M1 from six rats. (D, E) DEX‐induced coherograms changes between S1 and M1 in different frequency bands. *p < 0.05.

To further examine how the pattern of functional connectivity changes between S1 and M1 after DEX injection. The LFP correlogram between S1 and M1 was plotted (Figure 3). As shown in Figure 2E, although DEX‐induced power increase lays across a wide frequency range (1–25 Hz). The oscillatory coherence between S1 and M1 increased only in delta and low alpha band (Figure 3B–E).

### 3.3 Single Neuron Spiking Activities in S1 and M1 Correlated With DEX‐Induced Increase in Power Spectra of LFPs Differentially

**FIGURE 4:**
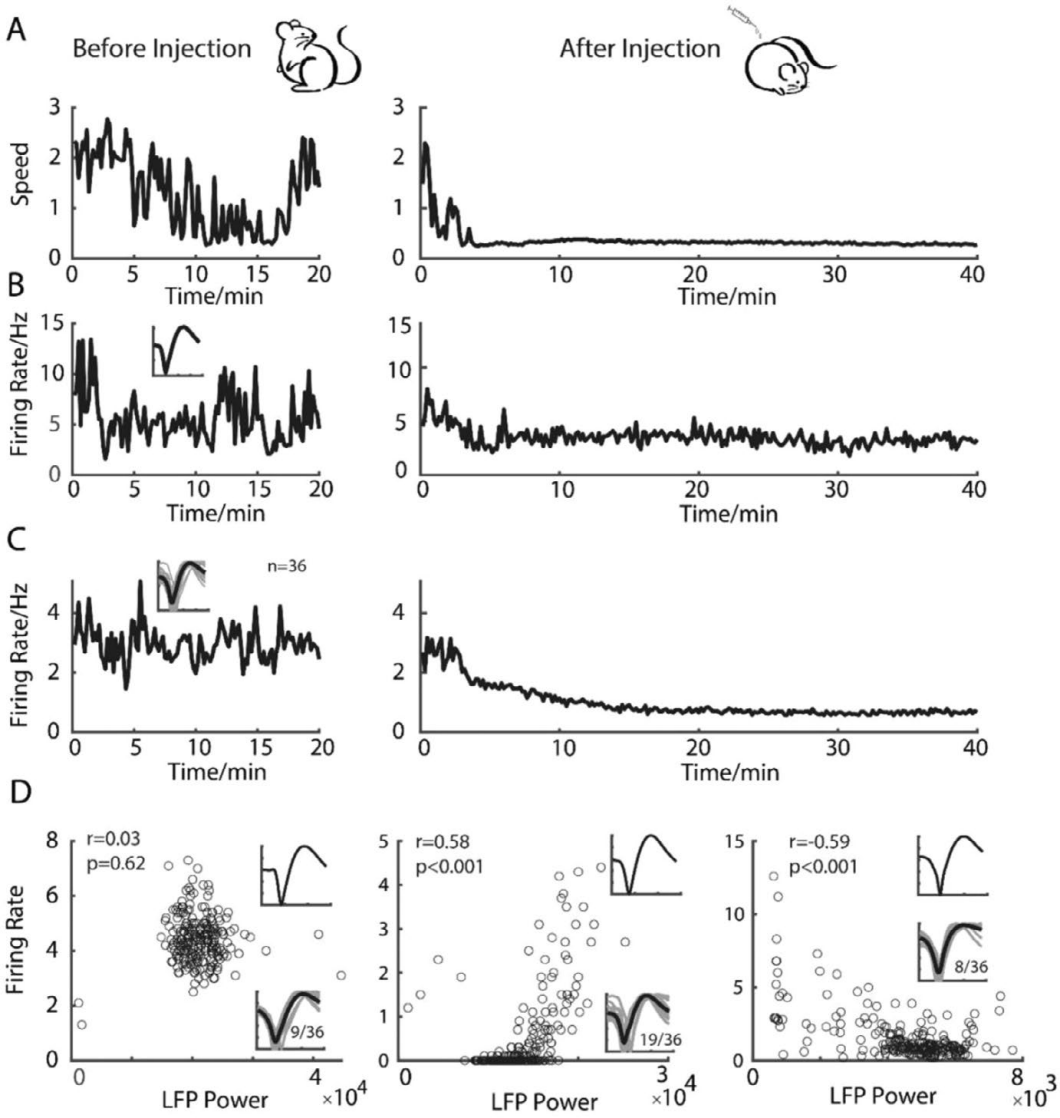
Correlation between the firing of individual neurons and the LFP activity in S1 and M1. (A) A representative of the locomotor activity before and after DEX injection. (B) Corresponding firing rate of a representative neuron from S1 before and after DEX injection. (C) Firing rate change of all recorded neurons from S1 and M1 before and after DEX injection. (D) Categories of neurons depending on the correlation between their firing rates and LFP activity.

To examine whether single neural activities have a temporal relationship with the corresponding LFPs under DEX‐induced anesthesia, correlations between single unit firing and corresponding LFP activity were calculated. As shown in Figure 4B,C, DEX caused an overall decrease of firing rate in all recorded neurons from both S1 and M1. Approximately 53% of neurons showed positive correlation between firing rates and the power of the LFP oscillation (Figure 4D middle). In contrast, 25% of neurons exhibited a negative correlation between the firing rates and the power of LFP (Figure 4D right). The rest 22% of neurons showed no correlation between the firing rate and LFP oscillation (Figure 4D left).

### 3.4 DEX‐Induced Increase in Power Spectra of LFP Differs From That of NREM Sleep

**FIGURE 5:**
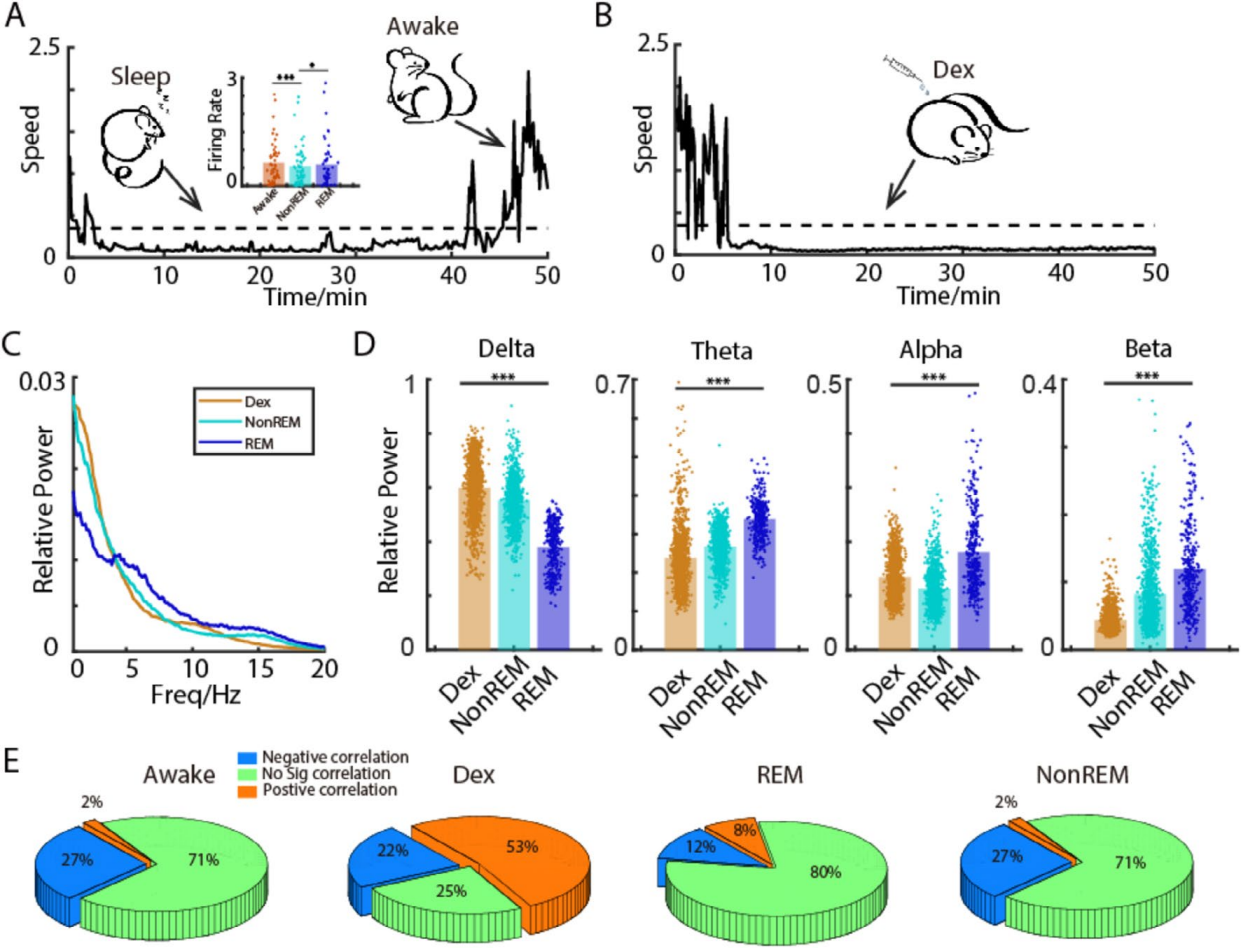
Comparison of the LFP power spectrum and the spike‐LFP coherence during natural sleep and DEX‐induced anesthesia. (A, B) A representative of rat activity during awake, sleep, and DEX‐induced anesthesia. (C) A representative quantification of the relative power spectrum during sleep and DEX‐induced anesthesia. (D) Quantification of the relative power spectrum during sleep and DEX‐induced anesthesia in different frequency bands. (E) Proportion of neurons that correlated negatively, positively, or do not correlate with the LFP activity during awake, sleep, and DEX‐induced anesthesia. ***p < 0.001 (Dex vs. REM).

**FIGURE 6:**
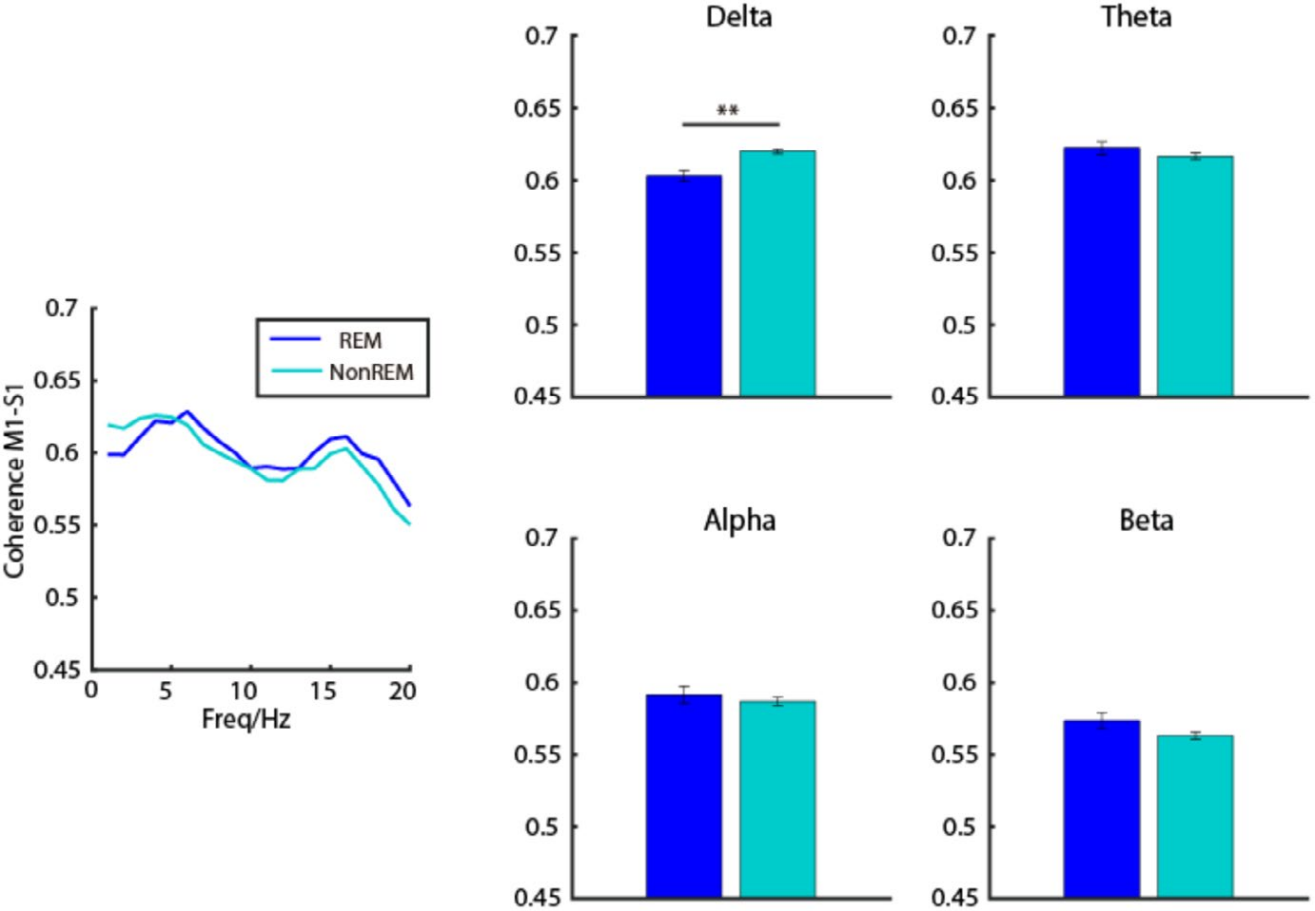
Comparison of the LFP coherence between M1 and S1 during REM and NREM sleep. **p < 0.01.

The neuronal signals during both sleep and DEX‐induced anesthesia were recorded. As shown in Figure 5A, sleep episodes were characterized by motionless with concurrent enhancement of slow wave activity in both S1 and M1. Among multiple sleep episodes, REM sleep fragments were characterized by significantly higher power in alpha band but relatively lower power in delta band than that in NREM sleep period (Figure 5C,D). Furthermore, higher LFP oscillatory coherence was seen in delta band between S1 and M1 during NREM sleep than those during REM sleep (Figure 6).

To test whether the pattern of DEX‐induced cortical activity is similar to that during NREM sleep as shown from EEG recordings, relative power spectrum of these two states was compared. As shown in Figure 5C,D, compared with NREM sleep, the spectrogram during DEX‐induced anesthesia exhibited relatively higher power in delta and alpha bands, but showed relatively lower power in theta and beta bands. Furthermore, to examine how the single neuron activity differs during sleep and DEX‐induced anesthesia, the temporal relationship between single unit spikes and the corresponding LFPs as analyzed. As seen in Figure 5E, the number of neurons correlate (either positively or negatively) to the LFPs during DEX‐induced anesthesia was much higher than that during sleep.

## 4 Discussion

In this study, the pattern of functional connectivity between S1 and M1 during DEX‐induced anesthesia and natural sleep was investigated. Results showed that DEX increases in power spectra of a relatively wide band (1–25 Hz). A significant increase in LFP coherence between S1 and M1 was found in the delta and low alpha band. DEX decreased the firing frequency of neurons in S1 and M1. However, neuronal spiking can be positively or negatively correlated with the power of LFP oscillation. Similarities were found between DEX‐induced anesthesia and natural sleep, while the distinct pattern of spike‐field correlation indicates the two states are modulated differently.

An invariant set of changes takes place during DEX‐induced anesthesia is enhanced low frequency oscillations (0–5 Hz) across entire scalp. This enhancement was mainly revealed by scalp EEG recordings in both humans [19, 23, 47, 48, 49, 50, 51, 52] and laboratory animals [53, 54, 55]. However, in the relatively higher frequency band (> 5 Hz), the pattern of oscillatory activity varies in different cortical areas, for example, frontal lobe exhibited higher power spectrum than occipital lobe in low beta band but lower power spectrum in high beta band [19, 47, 51, 56]. The signatures of DEX‐induced functional connectivity among different regions were examined by measuring the EEG coherence. Akeju and colleagues have reported that the theta and spindle oscillations were globally coherent under DEX‐induced anesthesia [47]. Although another study showed that compared with the baseline, DEX increased coherence in the delta, theta, and alpha bands and decreased coherence in the slow‐wave frequency band between the frontal region of the left and right hemisphere [19].

Studies of general anesthesia have proposed that loss of information processing capability in the cortex is resulted by enhanced regional synchrony while depressed long‐range correlations [57, 58, 59, 60, 61, 62]. Largely because the signals collected by EEG recordings can only provide measures of cortical dynamics at large spatial scales [63, 64]. It was still largely unknown how the pattern of cortical activity alters in certain cortical regions under DEX‐induced anesthesia let alone the changes of functional connectivity in specific neural circuits. In the present study, multichannel microelectrodes were pre‐planted into S1 and M1, respectively, to measure both LFPs and single neuron activity during awake, sleep, and under DEX‐induced anesthesia. To our knowledge, this is the first report that investigated the cortical functional connectivity between defined areas during sleep and DEX‐induced anesthesia. Consistent with the results from previous studies, DEX induced significant increase in power spectra of cortical slow wave oscillations in both S1 and M1. Furthermore, although there is no significant difference in terms of the power spectrum of S1 and M1, DEX resulted in significant increase in LFP coherence between S1 and M1 only seen in delta and low alpha bands.

Current theories proposed that delta activity originated in neurons in deep cortical layers [65, 66, 67, 68]. In the present study, DEX induced a significant increase in delta band LFP coherence between S1 and M1. This increase in delta band LFP coherence may reflect the extent of hyperpolarization or inhibition of cortical neurons, resulting in dedifferentiation of neural activity in S1 and M1. On the other hand, alpha band activity is thought to originate from “pacemaker neurons” throughout the thalamus or dipole layer in the widely distributed cortical centers. Here, DEX‐induced enhancement of alpha band coherence between S1 and M1 may indicate that thalamic functional connectivity with the cortex is not disturbed which is consistent with the previous result obtained by fMRI in humans [49].

Slow oscillations have been proposed as a shared feature for unconsciousness during sleep and DEX‐induced anesthesia [19, 69, 70, 71]. Earlier experimental evidence supports the notion that DEX targets the NREM sleep‐promoting pathways to induce its sedative effect [72]. The analysis of the EEG features during NREM sleep and DEX‐induced anesthesia revealed closely resembling cortical activity patterns [23]. In the present study, by comparing the relative power spectrum in S1 and M1 during natural NREM sleep and DEX‐induced anesthesia, significant differences in terms of the pattern of cortical activity were found between these two states. As mentioned earlier, relatively higher delta and alpha power during anesthesia may indicate that DEX exerted stronger inhibitory effect on the deep layer cortical neurons than the intrinsic sleep promoting factors such as epinephrine, adenosine. And the relatively higher power spectrum in theta and beta bands may indicate less cortical network inhibition during natural sleep.

LFP is seen as a temporal sequence of synchronized activity of a certain proportion of related neurons [73, 74, 75, 76, 77, 78, 79]. At the neuronal level, slow wave oscillations have been proposed to be associated with an alternation between ON states where neurons are able to fire and OFF states where neurons are silent [15, 19, 80, 81]. During stages 3 and 4 NREM sleep, also known as slow‐wave sleep [82], one of the prominent characteristics is the low neural firing rates but high synchrony with LFP oscillation which reflects slow oscillations between cortical down and up states [83, 84]. In the present study, DEX induced overall decrease of the firing rates in all the recorded units, further analysis revealed that half of these units exhibited positive correlation with the slow wave oscillations and a quarter of these units showed negative correlation with the slow wave oscillations. Meanwhile, a quarter of these units exhibited no correlation with the slow wave oscillations. In summary, the present results indicate that DEX and NREM sleep regulating cortical pathways may be vastly distinct.

Distinguishing dexmedetomidine‐induced anesthesia from natural sleep using neural oscillations has meaningful implications for both clinical practice and neuroscience research. Identifying unique oscillatory signatures associated with dexmedetomidine‐induced anesthesia could enable clinicians to monitor sedation depth more precisely, especially for patients requiring light sedation, such as during intensive care. By comparing oscillations between dexmedetomidine sedation and natural sleep, researchers can optimize anesthesia protocols to reduce cognitive side effects and facilitate faster recovery times. Analyzing differences in neural oscillations during dexmedetomidine anesthesia versus sleep contributes to a better understanding of how these states modulate consciousness. This knowledge may help researchers map how specific oscillations correlate with awareness, which could benefit studies on disorders such as coma or locked‐in syndrome. In summary, distinguishing dexmedetomidine anesthesia from sleep at the neural level could optimize sedation practices, improve patient outcomes, and deepen understanding of brain function across different conscious states.

## Author Contributions

D.G., C.H., and Z.M. designed the study. D.G. conducted the experiments. C.H., and D.G. analyzed data. D.G., C.H., C.L., and Z.M. contributed to the interpretation of results, D.G., C.L., and Z.M. wrote the manuscript. All authors contributed by editing the manuscript intensively. Z.M. supervised the study.

## Ethics Statement

All surgical and experimental protocols were approved by the Institutional Animal Care and Use Committees at the Shenzhen Institute of Advanced Technology, Chinese Academy of Sciences.

## Conflicts of Interest

The authors declare no conflicts of interest.

## Supporting information


Figures S1–S2.

## Data Availability

The data that support the findings of this study are available from the corresponding author upon reasonable request.
