# Supplementary material for: Neural Oscillations in the Somatosensory and Motor Cortex Distinguish Dexmedetomidine‐Induced Anesthesia and Sleep in Rats
Source: CNS Neurosci Ther. 2025 Feb 18;31(2):e70262. doi: 10.1111/cns.70262 (PMC11833454; doi:10.1111/cns.70262)
Supplement: Supplementary file 1 — Figures S1–S2. [file CNS-31-e70262-s001.docx]

**
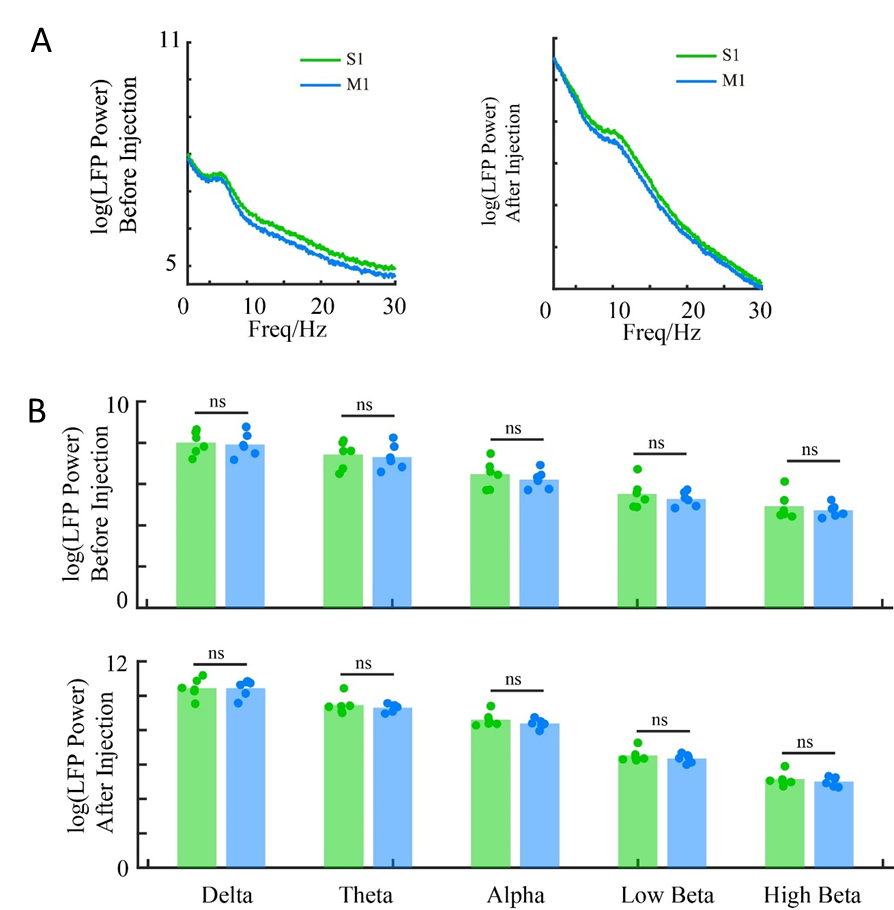
**

**Supplementary Figure 1. Spectrograms of S1 and M1 from 6 rats before and after DEX injection.** (A) A comparison of LFP activity in S1 and M1 before and after DEX injection in terms of its power magnitude. (B) Statistics of the LFP power magnitude in different bands in S1 and M1 after DEX injection. ns, no significant difference.


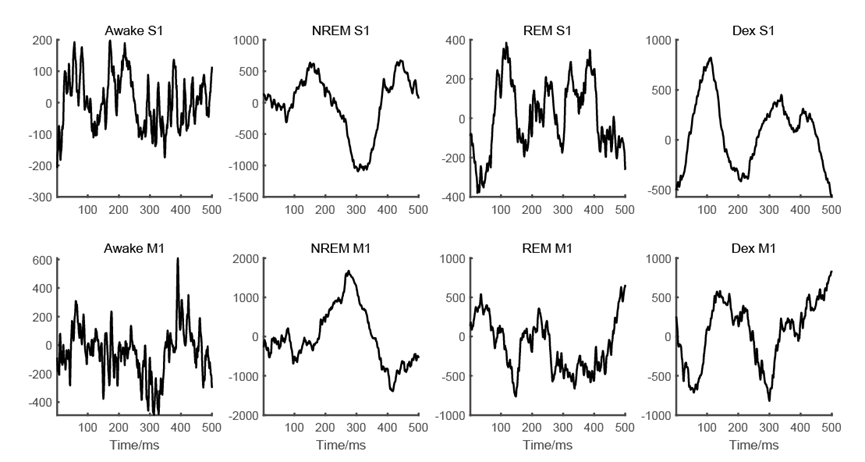


**Supplementary Figure 2. Examples of LFPs during wakefulness, NREM sleep, REM sleep and DEX-induced anesthesia.**
